# Reasons for the Intention to Refuse COVID-19 Vaccination and Their Association with Preferred Sources of Information in a Nationwide, Population-Based Sample in Italy, before COVID-19 Vaccines Roll Out

**DOI:** 10.3390/vaccines10060913

**Published:** 2022-06-08

**Authors:** Marco Del Riccio, Angela Bechini, Primo Buscemi, Paolo Bonanni, on behalf of the Working Group DHS, Sara Boccalini

**Affiliations:** 1 Medical Specialization School in Hygiene, Preventive Medicine and Public Health, University of Florence, 50134 Firenze, Italy; primo.buscemi@unifi.it (P.B.); duccio.giorgetti@unifi.it; 2 Department of Health Sciences, University of Florence, 50134 Firenze, Italy; angela.bechini@unifi.it (A.B.); paolo.bonanni@unifi.it (P.B.); sara.boccalini@unifi.it (S.B.)

**Keywords:** COVID-19, vaccines, hesitancy, sources of information, social media, internet

## Abstract

Sources of information on health and vaccines such as social media, online forums, televisions, and newspapers contributed to the spread of information related to COVID-19 and, in some cases, misinformation and vaccine hesitancy. Understanding what can influence the intention to refuse COVID-19 vaccination may help to plan future public health strategies aimed at increasing vaccination coverage. This study aimed to assess the reasons for the intention to refuse the COVID-19 vaccines and the possible association between these reasons and the preferred sources of information on vaccines. An anonymous online survey was shared among the general adult population living in Italy. Only participants aged 18 or older and living in Italy were considered eligible. The questionnaires that reported the intention to refuse COVID-19 vaccination were analyzed. A total of 677 participants (from 7563 valid questionnaires) reported the intention to refuse to vaccinate against COVID-19. Most of them used search engines (*n* = 390, 57.6%) to seek information about vaccines, while the fear of adverse reactions to the COVID-19 vaccine was the most mentioned reason for being unwilling to get vaccinated (*n* = 402, 59.4%). These data may be important to build new knowledge on the impact that different sources of information can have on the willingness to get vaccinated against COVID-19.

## 1. Introduction

Since early 2020, when COVID-19 appeared, the latest trends of the pandemic were shared with the population by traditional mass media (newspapers, televisions) and emerging information channels, such as social media [1]. The same happened when the first reports on the safety and the efficacy of the new vaccines against COVID-19 were released; many sources of information had an important role in influencing public opinion and determining whether one wanted to get vaccinated [2]. Recent studies have demonstrated how online forums, social media, online journals, televisions, and newspapers contributed to the spread of information related to COVID-19 and, in some cases, misinformation and vaccine hesitancy [3,4]. In particular, it is known that social media can enhance the susceptibility to the effects of misinformation and exacerbate vaccine hesitancy, decreasing compliance to guidance measures [5] by discrediting empirical data and scientific approaches and promoting personal, anecdotical tales [6]. In 2012, Nan and colleagues performed a controlled experiment and showed how those who were exposed to blogs that reported negative information about the HPV vaccine held more negative attitudes toward the vaccine and had reduced intentions to receive the vaccine [7].

The COVID-19 vaccines have a key role in fighting the pandemic; considering the need for vaccine uptake and the necessity to understand the different reasons that push people to refuse vaccination, we tried to describe the use of different channels for vaccine information and the reasons given by hesitant individuals regarding their intention to refuse vaccination, before COVID-19 vaccine availability.

Specifically, we aimed to assess the preferred sources of information on vaccines and the reasons for the intention to refuse the COVID-19 vaccines, before their availability on the market, in a nationwide sample of adults living in Italy, and the possible association between these reasons and the sources of information.

## 2. Materials and Methods

### 2.1. Study Design, Data Collection and Inclusion Criteria

An anonymous survey was shared online from 11 December to 15 December 2020 among the adult general population via the website “VaccinarSinToscana” [8], a platform developed by the Italian Society of Hygiene and Preventive Medicine at a regional level and through social platforms (Facebook, Instagram, WhatsApp, Telegram) that was launched on 21 March 2017 and had more than 65,000 visits during the first three years of activity [9]. Participation was voluntary and all subjects accepted to participate providing online informed consent. Inclusion criteria were: (i) being 18 or older; (ii) living in Italy; (iii) being able to fill in an online questionnaire in Italian. Participants younger than 18 years or living abroad were excluded, as well as incomplete questionnaires. 

### 2.2. Ethical Approval

The study was conducted according to the criteria set by the declaration of Helsinki and each subject signed informed consent before participating in the study. No ethical approval was required, as the participation was completely anonymous. 

### 2.3. Questionnaire

The questionnaire was designed by a group of senior and young researchers working in the field and it was aimed at exploring the intention to get vaccinated against COVID-19, potential determinants of the intention to get vaccinated and reasons brought by the participants to refuse vaccination, in case of refusal. The results that we are presenting in this manuscript come from a two-section questionnaire whose first section included the evaluation of the factors influencing COVID-19 vaccine acceptance and hesitancy among the respondents [10]. The second and last section of the questionnaire focused instead on the reasons to get vaccinated (or not) against COVID-19 and the preferred sources of information to learn more about vaccines. Sociodemographic and socioeconomic characteristics of the sample have been already described in prior work [10]. The questionnaire was designed and written in Italian.

### 2.4. Variables

Participants were first asked what sources of information they used to seek information on vaccine quality, vaccine safety and efficacy, and vaccine availability (possible answers were “Search Engines”, “Social Media”, “Television/Newspapers”, “Scientific Journals”, “Healthcare Professionals”, “I do not search information on vaccines”). They were allowed to select more than one answer. Next, the participants were asked about their intent to be vaccinated against COVID-19 (possible answers ranged from “1—surely not” to “5—surely yes”). Finally, those who answered “surely not” or “I do not think so” were asked a specific question about their reasons to avoid vaccination. Possible answers included: “COVID-19 does not represent a risk”; “I doubt this vaccine is effective”; “I fear adverse reactions”; “I do not trust vaccines”; “I do not know enough about this vaccine”; “I have been scared by the news I read on media and social media”. It was possible to select more than one answer (min: one answer; max: all answers). 

### 2.5. Analysis

Descriptive analyses were conducted on the group of participants that answered either “surely not” or “I do not think so” to the question “Will you get vaccinated against COVID-19 when a vaccine is approved, available, and recommended for you?”. Single and multiple logistic regression analyses were conducted to assess the association between each reason given for intention to refuse vaccination (dependent variables) and other variables: here, we only present the results of the multiple logistic regression analyses. Results are given as odds ratios (OR) with 95% confidence interval (95% CI). A *p*-value < 0.05 was considered significant. Statistical analyses were conducted using RStudio 1.2.5033/RStudio Team, 2019 (RStudio: Integrated Development for R. RStudio, Inc., Boston, MA, USA). 

## 3. Results

A total of 677 participants (from 7563 valid questionnaires) answered “surely not” or “I do not think so” when asked if they wanted to get vaccinated against COVID-19 in case a vaccine was approved and recommended for them. Table 1 and Table 2 show the summary statistics of the variables that were collected for this group. Most participants were female (*n* = 431, 63.7%), held a high school diploma (*n* = 282, 41.7%), and were not healthcare professionals (*n* = 435, 64.3%). The median age was 47.0 years (IQR 34.0–58.0). Among the preferred sources of information on vaccines, search engines (selected by *n* = 390, 57.6%) and doctor/healthcare professionals (*n* = 366, 54.1%) were the most selected options. When asked why they did not want to get vaccinated, most participants reported fear of adverse reactions (*n* = 402, 59.4%) and high distrust towards the upcoming COVID-19 vaccines (*n* = 332, 49%). 

According to multiple logistic regression models (Table 3), male subjects were more likely to report that COVID-19 did not represent a risk (OR 2.30, 95%CI 1.41–3.78, *p* < 0.001), as well as those who selected “social media” as a source of information (OR 2.01, 95%CI 1.03–3.79, *p* = 0.035). The latter were those who also had more chances to doubt COVID-19 vaccine efficacy (OR 1.93, 95%CI 1.19–3.13, *p* = 0.008) and distrust vaccines (OR 2.18, 95%CI 1.34–3.63, *p* = 0.002). In this sample, seeking information on internet search engines, asking a general practitioner or other healthcare professionals, and watching or reading the news were significant predictors of fearing adverse reactions and feeling illiterate about COVID-19 vaccines (Table 3).

## 4. Discussion

This study, conducted in December 2020 before any COVID-19 vaccine was available, aimed at assessing the preferred sources of information on vaccines and the reasons for the intention to refuse the COVID-19 vaccine in a nationwide sample of adults living in Italy, and the possible association between these reasons and the preferred sources. 

While COVID-19 vaccines are the most important intervention to control the pandemic and its effects on health, COVID-19 vaccine hesitancy is one of the most important limiting factors in the fight against the virus and has had a substantial negative impact on the pandemic trajectory [11]. Understanding the reasons to refuse to be vaccinated against COVID-19—and possible factors influencing them—are important to plan new strategies to increase vaccine uptake, as underlined by recent research [12]. 

In the present study, those who stated they would refuse to get vaccinated were 9% of the sample, which is the same proportion of adults that have not currently received any vaccine dose in Italy [13]. It is important to mention that the willingness (and the hesitance) to vaccinate at the time this study was conducted may have been strongly influenced by the lack of data and information on this vaccine’s safety and efficacy: no study had been published yet, with the first one being issued one week after that the closure of the data collection of this study [14]. In fact, even those who participated in this study and reported scientific journals as preferred sources of information on vaccines had doubts about vaccine effectiveness; moreover, 4 out of 10 participants reported that one of the main reasons for refusing to get vaccinated was the lack of enough knowledge about COVID-19 vaccines. Indeed, having appropriate, timely and reliable information on vaccine safety and effectiveness was recently highlighted as an important strategy to also promote influenza vaccination [15], especially considering that only a small fraction of the Italian population gets vaccinated against influenza (19.6% of the general population in 2019/2020, 23.6% in 2020/2021) [16] even in high-risk groups [17], being the only exception the elderly [18].

It should not be surprising that the internet is currently used as one of the main sources of information, considering that more than 50 million Italians are internet users, and the number is still growing (+66% in ten years) [19]. A recent institutional survey reported how four out of five internet users in Italy use the internet to find health information [20]. The findings of the current study are in alignment with other Italian and international studies: resorting to doctors and healthcare professionals is consistent with the findings of other studies [21,22] and highlights the key role that healthcare professionals can have in promoting appropriate vaccinations to people that doubt vaccine safety and fear of adverse reactions. This was indeed the most reported reason to refuse vaccination in our sample, and it was associated with being more likely to turn to a healthcare provider, as in other studies [23]. Despite the increasingly important role in spreading both accurate information and misinformation [24], social media was the least used source of information: this is not uncommon, as found and reported also by other authors [22,25]. Social media has been associated with vaccine hesitancy in the past. In particular, a relationship between the use of social media and doubts about vaccine safety, as well as disinformation campaigns and declining vaccination coverage, have been already observed [3,25]. In our sample, which only focused on hesitant subjects, it emerged that those who selected social media as one of the preferred sources of information were most likely to report a reduced risk perception towards COVID-19 as one of the main reasons to refuse vaccination, as well as doubts about vaccine efficacy. 

The association between the use of social media and low risk perception is currently debated, as different findings can be found in the literature [26,27,28]. This suggests that “social media” is a heterogeneous environment in which, depending on one’s attitude and expectations, the use of one or another platform and the exposure to diverse sources of information can have different impacts on the user. Considering this variability, it emerges how international and national institutions and public health institutes, that want to engage the public to provide accurate information on vaccination and reduce vaccine hesitancy, cannot ignore social media in their communication strategy. 

This study has several limitations. First, it involved a convenience sample of adults living in Italy and was only distributed to web users; therefore, it cannot be considered representative of the whole population, even if the number of hesitant individuals identified during the data collection in December 2020 is consistent with the actual Italian figures on COVID-19 vaccination uptake [13]. Moreover, it represents beliefs about COVID-19 vaccinations that were expressed before the beginning of the vaccination campaign. The opinions toward COVID-19 vaccines of the adult Italian population may have changed during the last year, considering the new information about COVID-19 vaccine effectiveness and safety evidence [29]: in fact, as the vaccination campaign had not started, no studies proving safety and effectiveness of COVID-19 vaccines had been officially published at the time this study was conducted. It must be acknowledged that, in order to complete the data collection before the start of the COVID-19 vaccination campaign, our questionnaire was not tested in a small sample before its use. Finally, we hypothesized and evaluated the relationship between the reasons for the intention to refuse COVID-19 vaccines and the preferred sources of information in our sample, but—despite being described in the literature—this association should be further tested with more specific, structured methods, to properly explore the true interactions between the two variables and the presence of other factors influencing or mediating it. 

Despite these limitations, we believe that our study adds data to the current knowledge on COVID-19 vaccine hesitancy and their information-seeking behavior, and it can be relevant to build new knowledge on the impact of the sources of information on the willingness to get vaccinated against COVID-19. 

## Figures and Tables

**Table 1:** Sociodemographic characteristics of those participants that declared they would surely or probably refuse COVID-19 vaccination (N = 677).

Characteristics	NA	N or Median	% or IQR
Age	0	47.0	38.0–55.0
*Sex*	0		
Male		246	36.3
Female		431	63.7
*Study title*	0		
Primary school		4	0.6
Secondary school		78	11.5
High school		282	41.7
Bachelor’s degree		118	26.0
Master’s degree		176	17.4
PhD		19	2.8
*Healthcare Professional*	0		
Yes		242	35.7
No		435	64.3

**Table 2:** Preferred sources of information on vaccines and reasons for the intention to refuse COVID-19 vaccination among those who declared they would surely or probably refuse COVID-19 vaccination (N = 677).

Questions and Answers	NA	N	% of Individuals That Selected Each Option (Multiple Choice)
*Q = Preferred source(s) to search information about vaccines quality, vaccines safety and efficacy, and vaccines availability*	0		
Search engines		390	57.6
Social media		84	12.4
Television/Newspapers		207	30.6
Scientific Journal		268	39.6
Doctor/Healthcare Professionals		366	54.1
I do not search information on COVID-19		35	5.2
*Q = Reasons to refuse COVID-19 vaccination*	0		
COVID-19 does not represent a risk		90	13.3
I doubt this vaccine is effective		249	36.8
I fear adverse reactions		402	59.4
I do not trust vaccines		332	49.0
I do not know enough about this vaccine		275	40.6
I have been scared by the news I read on media and social media		45	6.6

**Table 3:** Multiple regression models. Six different models are reported, considering each reason for the intention to refuse COVID-19 vaccination (columns) as the dependent variable and the other variables as potential predictors (rows).

	1. COVID-19 Does Not Represent a Risk		2. I Doubt This Vaccine is Effective		3. I Do Not Trust Vaccines		4. I Fear Adverse Reactions		5. I Do Not Know Enough about This Vaccine		6. I Have Been Scared by News I Read on Media and Social Media	
Characteristic	OR 95% CI	*p*-Value	OR 95% CI	*p*-Value	OR 95% CI	*p*-Value	OR 95% CI	*p*-Value	OR 95% CI	*p*-Value	OR 95% CI	*p*-Value
*Age*	0.95 (0.93, 0.97)	<0.001	0.99 (0.98, 1.01)	0.4	0.99 (0.98, 1.00)	0.2	0.99 (0.97, 1.00)	0.041	0.99 (0.98, 1.00)	0.10	1.01 (0.99, 1.04)	0.3
*Sex*												
Female	-		-		-		-		-		-	
Male	2.30 (1.41, 3.78)	<0.001	1.17 (0.83, 1.64)	0.4	1.12 (0.80, 1.56)	0.5	0.84 (0.60, 1.17)	0.3	0.90 (0.63, 1.27)	0.5	1.04 (0.52, 2.04)	>0.9
*Healthcare professional*												
No	-		-		-		-		-		-	
Yes	0.58 (0.32, 1.02)	0.065	0.94 (0.64, 1.37)	0.7	0.91 (0.63, 1.31)	0.6	0.85 (0.58, 1.24)	0.4	1.25 (0.85, 1.82)	0.3	1.64 (0.78, 3.40)	0.2
*Study title*												
Primary school	-		-		-		-		-		-	
Secondary school	0.00	>0.9	2.50 (0.27, 23.5)	0.4	5.72 (0.64, 124)	0.2	0.75 (0.08, 6.91)	0.8	3.23 (0.34, 30.6)	0.3	10.9 (0.86, 134)	0.053
High school	1.13 (0.50, 2.78)	0.8	1.10 (0.64, 1.93)	0.7	1.33 (0.79, 2.26)	0.3	0.79 (0.46, 1.36)	0.4	1.16 (0.67, 2.06)	0.6	0.75 (0.29, 2.19)	0.6
PhD	0.43 (0.02, 2.72)	0.4	0.91 (0.30, 2.61)	0.9	2.56 (0.90, 7.74)	0.083	2.02 (0.66, 7.02)	0.2	1.05 (0.32, 3.12)	>0.9	0.59 (0.03, 4.09)	0.6
Bachelor’s degree	1.21 (0.45, 3.38)	0.7	1.07 (0.56, 2.08)	0.8	1.33 (0.71, 2.51)	0.4	1.19 (0.62, 2.29)	0.6	1.98 (1.03, 3.87)	43	0.42 (0.11, 1.57)	0.2
Master’s degree	2.18 (0.93, 5.55)	0.085	0.84 (0.47, 1.55)	0.6	1.67 (0.95, 2.98)	0.077	0.73 (0.40, 1.30)	0.3	1.33 (0.73, 2.47)	0.4	0.43 (0.14, 1.42)	0.2
*Preferred source(s) to search information about vaccines quality, vaccines safety and efficacy, and vaccines availability*												
Search engines/internet	0.79 (0.48, 1.31)	0.4	1.27 (0.90, 1.78)	0.2	1.27 (0.92, 1.76)	0.15	1.39 (0.99, 1.94)	0.054	1.81 (1.29, 2.56)	<0.001	2.40 (1.17, 5.32)	0.022
Social media	2.01 (1.03, 3.79)	0.035	1.93 (1.19, 3.13)	0.008	2.18 (1.34, 3.63)	0.002	1.39 (0.84, 2.36)	0.2	0.94 (0.56, 1.56)	0.8	2.23 (0.97, 4.82)	0.049
Television/newspapers	0.67 (0.37, 1.20)	0.2	0.84 (0.57, 1.23)	0.4	1.07 (0.75, 1.55)	0.7	1.49 (1.02, 2.18)	0.039	1.94 (1.33, 2.84)	<0.001	1.26 (0.62, 2.50)	0.5
Doctor/healthcare professionals	1.46 (0.88, 2.47)	0.15	1.28 (0.91, 1.81)	0.15	1.12 (0.81, 1.55)	0.5	1.48 (1.06, 2.08)	0.021	2.18 (1.54, 3.09)	<0.001	2.47 (1.25, 5.20)	0.012
Scientific journals	1.65 (0.97, 2.82)	0.064	1.67 (1.16, 2.42)	0.006	1.14 (0.80, 1.64)	0.5	1.14 (0.79, 1.65)	0.5	1.36 (0.94, 1.98)	0.11	1.29 (0.63, 2.63)	0.5
I do not search information on COVID-19	1.24 (0.39, 3.51)	0.7	1.24 (0.53, 2.76)	0.6	1.34 (0.62, 2.92)	0.5	0.49 (0.21, 1.09)	0.085	1.99 (0.83, 4.57)	0.11	1.29 (0.07, 7.88)	0.8

## Data Availability

Data supporting reported results are available upon request to the corresponding author. Data were collected and managed in aggregated form according to European Union Regulation 2016/679 of European Parliament and the Italian Legislative Decree 2018/101.
